# Sodium-Glucose Cotransporter 2 Inhibitor Use and Outcomes in Transthyretin Amyloid Cardiomyopathy

**DOI:** 10.1016/j.jacadv.2024.101405

**Published:** 2024-11-15

**Authors:** Vikash Jaiswal, Muhammad Hanif, Yusra Mashkoor, Akash Jaiswal, Tanisha Prasad, Kripa Rajak, Muhammad Shahzeb Khan, Robert J. Mentz, Gregg C. Fonarow

**Affiliations:** a Department of Cardiovascular Research, Larkin Community Hospital, South Miami, Florida, United States; b Department of Internal Medicine, SUNY Upstate Medical University, Syracuse, New York, USA; c Department of Internal Medicine, Dow University of Health Sciences, Karachi, Pakistan; d Department of Geriatric Medicine, All India Institute of Medical Science, New Delhi, India; e Department of Geriatric Medicine, Fortis Memorial Research Institute, Gurgaon, India; f Royal College of Surgeons in Ireland, University of Medicine and Health Science, Dublin, Ireland; g Department of Cardiology, University of Missouri, Missouri, United States; h Department of Medicine, Baylor School of Medicine, Temple, Texas, United States; i Division of Cardiology, Heart Hospital Plano, Plano, Texas, United States; j Department of Cardiology, Baylor Scott and White Research Institute, Texas, United States; k Division of Cardiology, Duke University School of Medicine, Duke Clinical Research Institute, Durham, North Carolina, USA; l Ahmanson-UCLA Cardiomyopathy Center, Ronald Reagan UCLA Medical Center, Los Angeles, California, USA

**Keywords:** amyloidosis, outcomes, SGLT2 inhibitors, transthyretin amyloid cardiomyopathy

Transthyretin amyloid cardiomyopathy (ATTR-CM) is caused by myocardial deposition of misfolded transthyretin protein-forming amyloid fibrils which can result in symptomatic heart failure (HF).^1^ The only medication currently approved by the U.S. Food and Drug Administration for the treatment of ATTR-CM is tafamidis which has been shown to reduce cardiovascular-related hospitalization and all-cause mortality (ACM).^1^

**What is the clinical question being addressed?**

What is the effectiveness of sodium-glucose cotransporter 2 inhibitor among transthyretin amyloid cardiomyopathy patients ?

**What is the main finding?**

The use of SGLT2i is associated with a significant reduction in the risk of all-cause mortality, major adverse cardiovascular events (composite of acute myocardial infarction, ischemic stroke, hemorrhagic stroke, and cardiac arrest), and ischemic stroke in patients with ATTR-CM.

Sodium-glucose cotransporter 2 inhibitor (SGLT2i) has emerged as a cardio-kidney-metabolic drug in recent years that reduces HF hospitalization, improves cardiac function, and reduces the risk of chronic kidney disease progression.^2^ However, the efficacy of this medication class among patients with ATTR-CM has not been well established. Therefore, the present study aims to investigate the effectiveness of SGLT2i among patients with ATTR-CM.

The TriNeTX Global Collaborative Network research database was used to identify patients aged ≥18 years of age from January 2000 to April 2023. Patients were categorized into 2 groups, one with ATTR-CM on SGLT2i and a control group with ATTR-CM without SGLT2i. We used the International Classification of Diseases-10th Revision-Clinical Modification (ICD-10-CM) codes to identify comorbidities. ATTR-CM was identified using ICD-10-CM: E85.4. Patients were followed at 1 month, 1 year, and 3 years. Additional information about this database has been previously published.^3^ Propensity score matching (PSM) (1:1) was performed on age, sex, race, body mass index, hypertension, diabetes mellitus, chronic kidney disease, smoking status, hemoglobin level, low-density lipid level, left ventricular ejection fraction, NYHA functional class of heart failure, and various drugs including tafamidis, angiotensin-converting enzyme inhibitors, angiotensin receptor blockers, angiotensin receptor/neprilysin inhibitor, beta-blockers, diuretics, spironolactone, eplerenone, and statins. Primary outcome was ACM, while secondary outcomes were major adverse cardiovascular events (MACE), ischemic stroke, HF, atrial fibrillation (AF), and ventricular tachycardia (VT). All the outcomes were analyzed after 1 day of index event, ie, after starting SGLT2i and within 1 month, 1 year, and 3 years. Patients having outcome of interest prior to the window period were excluded from the final analysis. ICD-10-CM codes were used for all the outcomes of interest.

The balance of categorical and continuous covariates was assessed with the standardized mean difference, which is the difference in means or proportions over the pooled SD. Covariates were considered balanced in both groups when the standardized mean difference was <0.1. Relative risk (RR) along with 95% CI and *P* value were reported. All significance tests were 2-tailed and considered significant when *P* < 0.05. Statistical analyses were performed with the use of the TriNetX online platform.^3^

A total of 2,417 patients with SGLT2i and 32,860 without SGLT2i were analyzed among patients with ATTR-CM. After 1:1 PSM, 2,153 patients in the SGLT2i and 2,153 patients in the control group were included. The study population had a mean age of 74.3 ± 12.5 years, with 30.7% females and 23.8% Black individuals. The mean left ventricular ejection fraction was 50.9% ± 16.1% (Table 1). PSM analysis showed that SGLT2-inhibitors in patients with ATTR-CM were significantly associated with lower risk of ACM at 1 month (RR: 0.29 [95% CI: 0.19-0.44], *P* < 0.001), and at 1 year (RR: 0.51 [95% CI: 0.43-0.60], *P* < 0.001) compared with the control group. A similar trend was observed, with a significant reduction in the risk of MACE and ischemic stroke at 1 month (MACE, RR: 0.36 [95% CI: 0.20-0.67], *P* < 0.01; ischemic stroke, RR: 0.44 [95% CI: 0.21-0.95], *P* = 0.03), and at 1 year (MACE, RR: 0.55 [95% CI: 0.42-0.74], *P* < 0.01; ischemic stroke, RR: 0.64 [95% CI: 0.44-0.92], *P* = 0.01). However, the risk of HF (RR: 0.89 [95% CI: 0.64-1.23], *P* = 0.494), AF (RR: 0.91 [95% CI: 0.98-1.22], *P* = 0.547), and VT (RR: 1.10 [95% CI: 0.78-1.54], *P* = 0.572) at 1 year was comparable between the SGLT2i group and control group among patients with ATTR-CM. A similar trend in the outcomes was observed at 3-year follow-up (Table 1).

**Table 1:** Comparison of Baseline Characteristics and Clinical Outcomes Before and After PSM

Before PSM					After Propensity Score Matching PSM			
	Transthyretin Amyloid Cardiomyopathy With SGLT2i (n = 2,417)	Transthyretin Amyloid Cardiomyopathy Without SGLT2i (n = 32,860)	*P* Value	Std. Diff.	Transthyretin Amyloid Cardiomyopathy With SGLT2i (n = 2,153)	Transthyretin Amyloid Cardiomyopathy Without SGLT2i (n = 2,153)	*P* Value	Std. Diff
Age, y	73.8 ± 11.1	75.3 ± 12.6	<0.001	0.130	74.2 ± 10.8	74.4 ± 14.1	0.569	0.017
Age at index, y	72.0 ± 11.4	70.8 ± 13.0	<0.001	0.097	72.4 ± 11.1	72.6 ± 14.6	0.574	0.017
Sex								
Male	1,584	17,577	<0.001	0.247	1,392	1,401	0.774	0.009
Female	727	14,025	<0.001	0.264	664	657	0.817	0.007
Race								
White	1,303	19,555	<0.001	0.113	1,171	1,159	0.714	0.011
African American	590	4,947	<0.001	0.237	513	512	0.971	0.001
Asian	88	1,490	<0.001	0.045	80	88	0.529	0.019
Comorbidity								
Hypertension	2,123	17,227	<0.001	0.839	1,872	1,901	0.180	0.041
Diabetes mellitus	1,355	6,717	<0.001	0.788	1,149	1,172	0.482	0.021
Chronic kidney disease	1,252	7,337	<0.001	0.641	1,074	1,074	1.000	<0.001
Smoking	783	4,676	<0.001	0.440	661	641	0.507	0.020
Body mass index, kg/m^2^	29.3 ± 6.8	27.5 ± 6.5	<0.001	0.272	29.2 ± 6.7	28.6 ± 7.1	0.016	0.082
Medications								
Beta-blockers	1,910	12,892	<0.001	0.885	1,673	1,676	0.912	0.003
ARBs	1,270	5,631	<0.001	0.800	1,077	1,067	0.761	0.009
ACEi	1,132	7,489	<0.001	0.522	982	978	0.903	0.004
ARNI	407	421	<0.001	0.545	236	229	0.731	0.010
Statin	1,226	7,328	<0.001	0.635	1,040	1,060	0.801	0.007
Diuretics	2,143	12,543	<0.001	1.231	1,880	1,916	0.090	0.052
Spironolactone	1,216	2,822	<0.001	0.940	754	753	0.533	0.020
Eplerenone	163	196	<0.001	0.315	65	67	0.859	0.006
Tafamidis	642	419	<0.001	0.720	281	241	0.062	0.057
Heart function								
LVEF, %	49.2 ± 16.5	54.4 ± 14.8	<0.001	0.332	50.5 ± 16.0	51.5 ± 16.3	0.257	0.067
≥50%	565	2,703	<0.001	0.425	465	469	0.882	0.005
41%-50%	265	680	<0.001	0.366	189	192	0.872	0.005
≤40%	252	620	<0.001	0.361	180	177	0.868	0.005
NYHA functional class	2.5 ± 0.6	2.7 ± 0.8	0.125	0.215	2.5 ± 0.6	2.5 ± 0.8	0.894	0.027

	Clinical Outcomes Before and After PSM After 1-Month, 1-and 3-Year Follow-Ups			
	After 1-Month Follow-Up			
	RR Before PSM	*P* Value	RR After PSM	*P* Value
All-cause mortality	0.27 (95% CI: 0.19-0.38), (34 vs 1,537)	**<0.001**	0.29 (95% CI: 0.19-0.44), (27 vs 93)	**<0.001**
MACE	0.56 (95% CI: 0.37-0.84), (24 vs 537)	**0.005**	0.36 (95% CI: 0.20-0.67), (15 vs 35)	**0.001**
Ischemic stroke	0.32 (95% CI: 0.19-0.53), (15 vs 588)	**<0.001**	0.44 (95% CI: 0.21-0.95), (10 vs 21)	**0.031**
Heart failure	1.25 (95% CI: 0.77-2.00), (17 vs 634)	0.355	0.84 (95% CI: 0.45-1.58), (15 vs 26)	0.605
Atrial fibrillation	1.04 (95% CI: 0.68-1.59), (22 vs 421)	0.839	0.83 (95% CI: 0.45-1.54), (18 vs 23)	0.566
Ventricular tachycardia	1.15 (95% CI: 0.68-1.95), (15 vs 195)	0.586	0.98 (95% CI: 0.43-2.21), (11 vs 12)	0.961
After 1-y follow-up				
All-cause mortality	0.62 (95% CI: 0.55-0.70), (254 vs 5,004)	**<0.001**	0.51 (95% CI: 0.43-0.60), (191 vs 371)	**<0.001**
MACE	0.77 (95% CI: 0.64-0.93), (106 vs 1726)	**0.008**	0.55 (95% CI: 0.42-0.74), (74 vs 114)	**<0.001**
Ischemic stroke	0.63 (95% CI: 0.50-0.79), (74 vs 1,471)	**<0.001**	0.64 (95% CI: 0.44-0.92), (47 vs 69)	**0.016**
Heart failure	1.40 (95% CI: 1.09-1.80), (57 vs 1890)	**0.008**	0.89 (95% CI: 0.64-1.23), (51 vs 84)	0.494
Atrial fibrillation	1.46 (95% CI: 1.21-1.70), (108 vs 1,478)	**<0.001**	0.91 (95% CI: 0.68-1.22), (77 vs 90)	0.547
Ventricular tachycardia	1.77 (95% CI: 1.40-2.19), (93 vs 787)	**<0.001**	1.10 (95% CI: 0.78-1.54), (65 vs 63)	0.572
After 3-y follow-up				
All-cause mortality	0.63 (95% CI: 0.56-0.67), (409 vs 7,906)	**<0.001**	0.53 (95% CI: 0.46-0.60), (289 vs 540)	**<0.001**
MACE	0.78 (95% CI: 0.67-0.90), (163 vs 2,584)	**0.001**	0.64 (95% CI: 0.51-0.81), (113 vs 148)	**<0.001**
Ischemic stroke	0.56 (95% CI: 0.46-0.69), (96 vs 2078)	**<0.001**	0.51 (95% CI: 0.37-0.69), (60 vs 108)	**<0.001**
Heart failure	1.27 (95% CI: 1.02-1.57), (73 vs 2,677)	**0.031**	0.91 (95% CI: 0.68-1.22), (61 vs 101)	0.531
Atrial fibrillation	1.35 (95% CI: 1.16-1.56), (160 vs 2,325)	**<0.001**	0.91 (95% CI: 0.72-1.14), (115 vs 137)	0.434
Ventricular tachycardia	1.59 (95% CI: 1.33-1.89), (134 vs 1,234)	**<0.001**	0.86 (95% CI: 0.65-1.13), (87 vs 108)	0.303

Values are mean ± SD or % unless otherwise indicated. The **bold** values show significant association.

ACEi = angiotensin-converting enzyme inhibitors; ARB = angiotensin receptor blocker; ARNI = angiotensin receptor/neprilysin inhibitor; LVEF = left ventricular ejection fraction; MACE = major adverse cardiovascular events; PSM = propensity score matching; SGLT2i = sodium-glucose cotransporter 2 inhibitor.

This study shows that the use of SGLT2i was associated with reduced risk of ACM, MACE, and ischemic stroke at 1-month, 1-year, and 3-year follow-up periods. These benefits, however, did not extend to HF, AF, and VT risks, which were comparable between the SGLT2i and control groups.

A significant reduction in ACM and MACE was observed among patients treated with SGLT2i which is likely due to its multifaceted cardiovascular benefits.^2^^,^^4^ These include reduced myocardial inflammation and fibrosis, enhanced myocardial energy efficiency, and diuretic effects that alleviate fluid overload.^4^ Furthermore, the observed reduction in risk of ischemic stroke with SGLT2i use can be attributed to improved glycemic control and systemic vascular health.^4^ Unlike many traditional HF therapies, SGLT2i does not adversely affect hemodynamics in ATTR-CM, making it a potential ideal therapy for advanced disease stages where symptomatic hypotension is a concern.^1^^,^^4^ The lack of significant differences in the risk of HF, AF, and VT between the SGLT2i and control groups may be due to the complex nature of ATTR-CM, where persistent high filling pressures and amyloid deposition in the myocardium predispose patients to HF decompensation and arrhythmias, necessitating additional therapeutic strategies.^1^

In comparison to Porcari et al,^5^ this study offers advantages, including a larger and more diverse sample size, enhancing generalizability. It broadens the analysis by evaluating outcomes like ischemic stroke and AF, not emphasized by Porcari. Furthermore, it provides both short- and long-term follow-up data and employs rigorous statistical methods, including multiple covariates in PSM. Nonetheless, limitations exist, such as potential residual confounding, limited generalizability to other forms of cardiac amyloidosis, possible indication bias in SGLT2i treatment decisions, and unavailability of certain variables at baseline such as disease severity, and dose of loop diuretics.

In conclusion, SGLT2i is associated with reduced ACM, MACE, and ischemic stroke in patients with ATTR-CM, without increasing the risk of HF, AF, or VT. These findings are particularly encouraging given the emerging disease-modifying treatments for this condition, indicating that SGLT2i may effectively complement these new therapeutic options.

## Funding support and author disclosures

Dr Fonarow has done consulting for Abbott, Amgen, AstraZeneca, Bayer, Boehringer Ingelheim, Cytokinetics, Eli Lilly, Johnson & Johnson, Medtronic, Merck, Novartis, and Pfizer. All other authors have reported that they have no relationships relevant to the contents of this paper to disclose.
